# The In Vitro Impact of the Herbicide Roundup on Human Sperm Motility and Sperm Mitochondria

**DOI:** 10.3390/toxics6010002

**Published:** 2017-12-21

**Authors:** George Anifandis, George Amiridis, Konstantinos Dafopoulos, Alexandros Daponte, Eleni Dovolou, Eleftherios Gavriil, Vyron Gorgogietas, Elli Kachpani, Zissis Mamuris, Christina I. Messini, Katerina Vassiou, Anna-Maria G. Psarra

**Affiliations:** 1 Department of Obstetrics and Gynaecology, ART Unit, University of Thessaly, School of Health Sciences, Faculty of Medicine, 41222 Larissa, Greece; kdafop@med.uth.gr (K.D.); dapontea@otenet.gr (A.D.); ellikachpa@hotmail.com (E.K.); pireaschristina@gmail.com (C.I.M.); 2 Department Obstetrics and Reproduction, University of Thessaly, Veterinary Faculty, School of Health Sciences, 43100 Karditsa, Greece; gsamir@vet.uth.gr (G.A.); lena.dovolou@yahoo.gr (E.D.); 3 Department of Biochemistry and Biotechnology, University of Thessaly, School of Health Sciences, 41221 Larissa, Greece; elefthgavriil@gmail.com (E.G.); norivbio7@gmail.com (V.G.); zmamur@bio.uth.gr (Z.M.); 4 Department of Anatomy, University of Thessaly, School of Health Sciences, Faculty of Medicine, 41221 Larissa, Greece; avassiou@med.uth.gr

**Keywords:** herbicides, Roundup, glyphosate, sperm motility, mitochondria

## Abstract

Toxicants, such as herbicides, have been hypothesized to affect sperm parameters. The most common method of exposure to herbicides is through spraying or diet. The aim of the present study was to investigate the effect of direct exposure of sperm to 1 mg/L of the herbicide Roundup on sperm motility and mitochondrial integrity. Sperm samples from 66 healthy men who were seeking semen analysis were investigated after written informed consent was taken. Semen analysis was performed according to the World Health Organization guidelines (WHO, 2010). Mitochondrial integrity was assessed through mitochondrial staining using a mitochondria-specific dye, which is exclusively incorporated into functionally active mitochondria. A quantity of 1 mg/L of Roundup was found to exert a deleterious effect on sperm’s progressive motility, after 1 h of incubation (mean difference between treated and control samples = 11.2%) in comparison with the effect after three hours of incubation (mean difference = 6.33%, *p* < 0.05), while the relative incorporation of the mitochondrial dye in mitochondria of the mid-piece region of Roundup-treated spermatozoa was significantly reduced compared to relative controls at the first hour of incubation, indicating mitochondrial dysfunction by Roundup. Our results indicate that the direct exposure of semen samples to the active constituent of the herbicide Roundup at the relatively low concentration of 1 mg/L has adverse effects on sperm motility, and this may be related to the observed reduction in mitochondrial staining.

## 1. Introduction

Among the variety of toxicants that affect male fertility, fungicides, insecticides, and herbicides seem to be environmental factors that may have a negative impact on human sperm quality [1,2]. Many of these chemicals are endocrine disruptors; their bioaccumulation in humans may cause adverse health effects, especially for people living in regions in which outdoor spraying has been performed to protect crops from competing weeds. Concerning the herbicide Roundup (a branded herbicide produced by Monsanto), there are no data relative to the effect of the in vitro exposure of male reproductive fluids (semen plasma) to this herbicide, although in the last decade of research studies have focused on reproductive toxicity caused by Roundup. The active ingredient of Roundup is glyphosate ([*N*-(phosphonomethyl)-glycine], which acts as an inhibitor of the enzyme 5-enolpyruvylshikimate-3-phosphate synthase by interrupting the synthesis of essential aromatic amino acids in plants [3]. The extensive use of glyphosate, given that it poses a risk to reproductive health, is a controversial matter. There were no adverse effects of glyphosate on mammalian reproduction and prenatal development [4], while another study described the negative impact of glyphosate on frog and chicken development [5]. Although the effect of the herbicide Roundup on human male reproduction is not known, a meta-analysis revealed that exposure to glyphosate decreased sperm concentration in both mice and rats, indicating adverse effects on reproductive health [6]. Moreover, studies on zebrafish demonstrated the harmful effect of glyphosate on sperm motility, mitochondrial functionality, and sperm DNA integrity [7]. In a previous study it was demonstrated that glyphosate at a concentration of 5 mg/kg exerted deleterious effects on sperm quality in Wistar rats [8], while recently it was reported that sperm parameters such as motility and concentration represented the most sensitive parameters affected by glyphosate in live-bearing *Jenynsia multidentata* [9].

Since more research on Roundup exposure is needed, we undertook the present study aiming to investigate for the first time the possible impact of direct in vitro exposure of human sperm cells to the herbicide Roundup as regards specific sperm characteristics, focusing on sperm motility. The effect of the herbicide on mitochondrial integrity was also assessed. Thus, taking into account the observed correlation between sperm motility and mitochondrial functionality [10], we attempted to investigate whether the herbicide Roundup could affect sperm motility and whether this action is associated with mitochondrial dysfunction. 

## 2. Materials and Methods

### 2.1. Human Subjects

Sixty-six (66) healthy men seeking semen analysis volunteered for the study during 2015 and gave written informed consent, while Institutional Review Board approval of the study was also obtained. All men were living in Thessaly in Greece, which is an agricultural region. Sixty percent of the participants (40/66) were doing an office job, 22.7% (15/66) were temporarily unemployed, and 17.3% (11/66) were salesmen. Only 15% (10/66) were married, while 20% (2/10) of the married men had one child (boy).

### 2.2. Sperm Collection and Preparation

Sixty-six fresh semen samples were collected after 48 h to 96 h of abstinence and were allowed to liquefy at 37 °C for 15 min to 20 min. Semen analysis of each specimen was performed in terms of semen volume and sperm concentration determination in combination with the percentage of progressive motile (PRM), non-progressive motile (NPM) and immotile (IM) spermatozoa, according to WHO 2010 guidelines. Portions of 0.5 mL of the total volume of each specimen were centrifuged at 2000 rpm for 5 min and the supernatant from each sample was carefully discarded, while the pellet was resuspended in 1 mL of pre-warmed buffer solution (Gamete Buffer, William Cook Australia PTY LTD^®^, Brisbane, Australia) containing Roundup at a final concentration of 1 mg/L (corresponding to a glyphosate concentration of 0.36 mg/L). Although the effect of the herbicide is profound in high concentrations, we investigated in a time-dependent manner the impact of the minimum concentration, which has the maximum effect as a model of toxicity in male gametes. The selected dose of the 0.36 mg/L glyphosate through the 1 mg/L Roundup was lower than the mean blood glyphosate concentration found in mild–moderate cases of human Roundup intoxication; in the latter cases, it has been found to be approximately 61 mg/L [11], while Roundup is used in agricultural work at dilutions ranging from 10 g/L to 20 g/L. Before the selected concentration used in the present study, we preliminary exposed spermatozoa of five normal semen samples with two other concentrations of 1/10 and 10 times higher. The first concentration did not show any impact on the spermatozoa, while in the second we observed total immotility. An aliquot of each initial fresh semen sample was kept aside for subsequent sperm analysis and served as a control (C). After 1 and 3 h incubation, in the presence or absence of Roundup, at room temperature, sperm analyses were performed in both control and Roundup-treated semen samples. Both control and Roundup-treated samples were subjected to the same experimental procedure. All samples for the purpose of the experiment were normalized to 10^6^ sperm cells in order to avoid any bias relative to the specific concentration of Roundup to different sperm concentration. 

### 2.3. Mitochondrial Staining

More than 300 spermatozoa of both control and Roundup-treated samples were examined. Approximately one-tenth by volume of the Roundup-treated and the un-treated (control) samples were centrifuged at 2000 rpm for 5 min. The supernatant from each sample was carefully discarded and the pellet was re-suspended in pre-warmed Dulbecco’s Modified Eagle Medium (Life technologies corporation, Grand island, NY, USA), supplemented with 200 nM of Mitotracker Red CMXROS (CMX) mitochondrial dye. CMX is a commercial red-fluorescence dye purchased from Invitrogen (Life technologies corporation, Grand island, NY, USA) that stains mitochondria in live cells, and its accumulation is dependent upon membrane potential. Thus, CMX is incorporated mainly in functionally active mitochondria of live cells [12,13,14,15,16]. Ten to 20 μL of the CMX-treated cells were allowed to settle down in areas marked by a hydrophobic barrier pen, on polylysine coated glass slides, for 1 h at 37 °C in a humidified atmosphere. Subsequently, attached cells were washed with Phosphate Buffer Saline (PBS), fixed in Methanol (−20 °C) for 10 min [17], washed three to four times with PBS, and mounted in a polyvinyl alcohol-based anti-fading medium [18]. Specimens were observed with a Leica DM2000 (Leica, Heerbrugg, Switzerland) fluorescence microscope equipped with a 100 × 1.65 oil objective lens. Images were taken with the Optimos (Qimaging, Surrey, BC, Canada) camera (Bodossaki Foundation donation). Quantitative analysis of the incorporated CMX dye in mitochondria of the sperm midpiece was performed with the use of ImageJ v1.47 program (NIH, Bethesda, MD, USA).

### 2.4. Statistical Analysis

Demographic data, sperm characteristics (volume, concentration and motility) were normally distributed (one sample Kolmogorov–Smirnov test) and statistical analysis was performed both by the paired *t*-test and the non-parametric Wilcoxon–Mann–Whitney test. The level of 0.05 was used to determine statistical significance. Numeric values were expressed as mean ± SD. The statistical software package used was SPSS v.17.

## 3. Results

**Figure 1:**
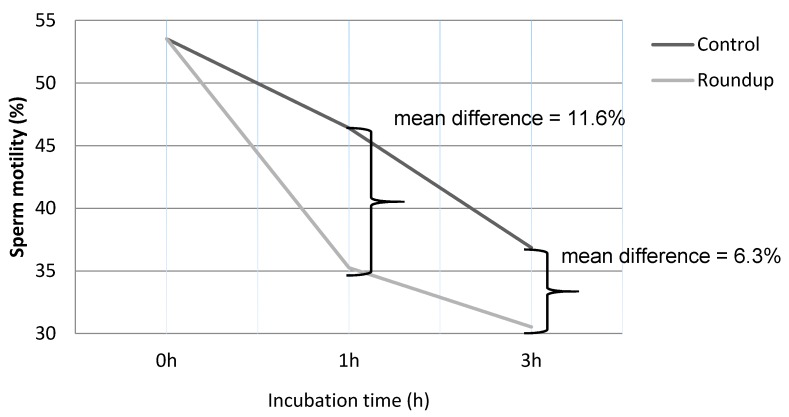
Schematic presentation of the percentage (%) of the progressive motility (PRM) between untreated (control) and the herbicide-(Roundup-) treated sperm samples. After 1 h of exposure to the Roundup, an approximately 11.16% reduction in motility was observed compared to control cells, whereas after 3 h incubation an approximately 6.33% decrease was observed, compared to controls (11.16% vs. 6.33%, *p* < 0.05).

The demographic data of all men studied are shown in Table 1. Mean sperm PRM of control (not Roundup-treated) cells (46.42 ± 16.19%, *p* < 0.05) was significantly reduced after 1 h of incubation compared to the PRM at zero time (53.54 ± 16.43%). PRM of Roundup-treated semen samples upon 1 h of incubation was 35.26 ± 15.21% and was significantly lower (*p* < 0.05) compared to the respective untreated (controls) semen samples at the same h. At 3 h post-incubation, the PRM of Roundup-treated semen samples was significantly lower in comparison to the respective controls (30.53 ± 11.67% vs. 36.86 ± 13.42%, *p* < 0.05) (Table 2). In Figure 1 the decline of PRM of both controls and Roundup-treated semen samples at 0, 1, and 3 h post-incubation is shown. As shown in Figure 1, after 1 h of exposure to Roundup, the rate of decline of PRM was more pronounced (11.16% decrease; 46.42 ± 16.19 vs. 35.26 ± 15.21%) compared to that observed after a further 2-h exposure (6.33% decrease; 30.53 ± 11.67% vs. 36.86 ± 13.42). 

### Mitochondrial Functionality

**Figure 2:**
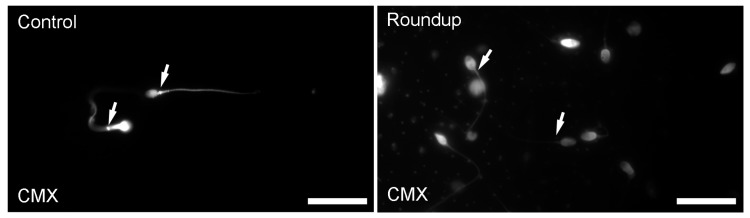
A representative image of mitochondrial staining in human spermatozoa exposed to Roundup. The exposure of sperm samples to Roundup for 1 h caused a reduction in the mitochondrial incorporation of the CMX dye compared to the controls (white arrows), indicating that Roundup caused impairment in mitochondria of the midpiece of human spermatozoa. CMX: mitochondria staining. Bars indicate 20 μm.

Exposure of sperm samples to Roundup reduced the mitochondrial incorporation of the CMX dye. In Figure 2, a representative image of mitochondrial staining of human spermatozoa exposed to Roundup is shown. Evaluation of the relative fluorescence intensity per unit area (RFU) of mitochondria in the mid-piece region of spermatozoa revealed a statistically significant reduction in the CMX incorporation in the Roundup-treated samples (0.66 ± 0.49) compared to controls (1.21 ± 0.95) (*p* < 0.05) (non-parametric Wilcoxon–Mann–Whitney test).

## 4. Discussion

In the present study, it is demonstrated for the first time that the herbicide Roundup has a direct impact on human sperm motility and sperm mitochondrial dysfunction. We used the parameter of motility because it is an in vitro study but also because progressive motility is the main aspect with respect to fertilization, while mitochondria activity may be used as a predictive factor for the motility of the spermatozoa. After 1 h exposure of sperm cells to 1 mg/L herbicide, a significant decrease in sperm progressive motility was observed which was accompanied by a significant reduction in the mitochondrial staining compared to control cells. Since mitochondrial staining with CMX dye is correlated with mitochondrial functionality, our data indicate that the progressive reduction in sperm motility is possibly correlated with mitochondrial dysfunction.

Roundup is widely used in agriculture worldwide, while glyphosate is its main active constituent. Glyphosate is known for its effect on plant physiology [3]. Interesting information about the impact of glyphosate on testicular functions is also available [19,20]. In a recent study it was found that acute Roundup exposure at low doses (0.036 g/L) induces oxidative stress, activating multiple stress-response pathways resulting in Sertoli cell death [17]. Sertoli cell death was associated with a significant increase in Ca^+2^ which led to a disruption of calcium homeostasis after acute exposure to glyphosate. A proposed mechanism for this action involves the activation of L-type voltage-dependent calcium channels, which in turn activates various protein kinases (PKC, PKA, and PI3K) and MAPK signaling pathways. In addition, the impact of glyphosate on human reproduction is proposed via its actions on aromatase activity and testosterone levels, acting as an endocrine disruptor [21,22]. A significant reduction in serum testosterone concentration and testicular morphology in male Wistar rats treated with Roundup has been documented [23], while the German Federal Institute for Occupational Safety and Health [24] provided evidence that there is no adverse reproductive effect on rats exposed to high doses of glyphosate. Recently, a report by the European Food Safety Authority showed that glyphosate is unlikely to be toxic to reproduction or development [25]. This notion is in line with results found during the systematic review of Araujo and colleagues, in which it was shown that glyphosate is not a human reproductive and developmental toxicant [26], since no significant associations of both paternal and maternal exposure to glyphosate with birth malformations were demonstrated. This study had many limitations. Moreover, in a study conducted in Colombia that evaluated the environmental and human health risks of aerially applied glyphosate, it was found that glyphosate did not pose any significant risk to human health, while the environmental risk was judged to be negligible [27,28]. Lastly, it was found that even in highly exposed populations the exposure was within the regulatory limits [29].

To the best of our knowledge, there are no available measurements of glyphosate concentration in seminal plasma. However, a higher plasma concentration of glyphosate causes mild intoxication [11]. Moreover, apart from glyphosate there are also other constituents of the herbicide that are likely toxic. The mechanisms of toxicity of Roundup are complicated since the unspecified inert ingredients contribute to its toxicity. Nevertheless, it has been recently proposed that Roundup might cause reproductive toxicity in male albino rats and reproductive dysfunction in Wistar rats [30,31]. Finally, in an experimental pilot study we undertook comparing the impact of Roundup with glyphosate alone, we observed that the impact of both herbicides on spermatozoa was similar, which means that the effect was most likely due to glyphosate and not to the surfactant.

The in vitro impact of Roundup on human male gametes has not been investigated. Taking into account the relationship between sperm motility, mitochondrial function, and steroids’ effect on mitochondrial function [32,33,34], we attempted to investigate the impact of exposure of Roundup on spermatozoa motility and mitochondrial staining using a specific mitochondrial dye whose intensity, according to the literature, is correlated with mitochondrial integrity and functionality [12,13,14,15,16]. Similar results have been observed for the pesticide DDT on sperm parameters in a dose-dependent manner [35,36], indicating that men who are exposed to high concentrations of DDT are at risk of developing infertility problems. The association between sperm immobility and mitochondrial functionality has also been demonstrated in spermatozoa after exposure to 1,1-dichloro-2,2-bis(4-chlorophenyl)ethylene], the main metabolite of DDT, p,p-DDE. In that case, a concentration-dependent effect on sperm motility and mitochondrial functionality was observed when applying flow cytometry [37]. In the present study, sperm motility was significantly decreased after 1 h of exposure to Roundup. This observation, in combination with the Roundup-induced reduction in mitochondrial staining, indicates that upon Roundup exposure the main male reproductive contents, spermatozoa, are subjected to deteriorating effects including mitochondrial dysfunction. Because of the crucial role of mitochondria in steroid hormone biosynthesis, energy production, reactive oxygen species generation, and integration of steroid receptors functions [38], any dysregulation of their function could affect sperm motility and cellular fate, as regards survival or induction of apoptosis [39]. Moreover, there are several studies indicating that glyphosate induces oxidative stress and damages unsaturated fatty acids [9,40,41,42]. 

In this study, the profound effect of Roundup on sperm immotility has been observed, which is also possibly correlated with the Roundup-induced mitochondrial impairment. Mitochondrial dysfunction could possibly cause an increase in mitochondrial-dependent apoptosis. The toxic effect of Roundup on spermatozoa is possibly mediated through the induction of oxidative stress and mitochondrial apoptotic signals production. The exact biochemical pathways affected by Roundup and/or its metabolites remain to be elucidated. In a pilot experimental study, we measured the DNA fragmentation index, with the use of the Halosperm kit, in Roundup-treated and untreated spermatozoa and found comparable results in DNA fragmentation between the two groups (data not shown). Lopes and colleagues [7] found that fish sperm DNA integrity was significantly reduced after exposure of spermatozoa to Roundup. Recently, it was observed that glyphosate alone has low toxicity on male reproductive system of rats [43]. The reason for mentioning DNA integrity is that it is a significant biomarker of male fertility potential, since sperm DNA fragmentation has been associated with sub-fertility [44,45]. Nevertheless, it cannot be ruled out that sperm DNA fragmentation is related to sperm staining patterns [46] and the methods applied [47].

To conclude, in the present study the direct in vitro impact of Roundup on human sperm motility is demonstrated for the first time; this may be mediated through mitochondrial deregulation. It is suggested that, at the particular dose used in the present study, Roundup can cause male sub-fertility, but more studies are needed to delineate the exact biochemical mechanism of action of Roundup on sperm motility and mitochondrial impairment.

## Figures and Tables

**Table 1:** Demographic data (mean ± SD) of all men studied.

Variable	Value
No of samples	66
Age (years)	40.21 ± 6.1
BMI (kg/mL)	28.65 ± 3.47
Semen Volume (mL)	3.33 ± 1.42
Sperm concentration (10^6^/mL)	49.61 ± 50.9

**Table 2:** Percentage of sperm motility in untreated (control) and Roundup-treated 66 semen samples at 0, 1, and 3 h post-incubation.

	PRM (%)	NPM (%)	IM (%)
Control (0 h) ^a^	53.54 ± 16.43	13.57 ± 7.93	32.61 ± 15.85
Control (1 h) ^b^	46.42 ± 16.19	12.21 ± 9.22	41.35 ± 15.33
Roundup (1 h) ^c^	35.26 ± 15.21	10.17 ± 7.68	54.89 ± 17.42
Control (3 h) ^a^	36.86 ± 13.42	11.09 ± 9.87	52.05 ± 13.7
Roundup (3 h) ^c^	30.53 ± 11.67	9.17 ± 8.72	60.47 ± 15.33

Control vs. Roundup between 0 and 1st h: ^a^ vs. ^b,c^ and ^b^ vs. ^c^: *p* < 0.05 for PRM% and IM%; Control vs. Roundup at 3rd h: ^a^ vs. ^c^: *p* < 0.05 for PRM% and IM%.
